# Identification of Biomarkers Related to Immune Cell Infiltration in Hepatocellular Carcinoma Using Gene Co-Expression Network

**DOI:** 10.3389/pore.2021.601693

**Published:** 2021-04-02

**Authors:** Wanbang Zhou, Yiyang Chen, Ruixing Luo, Zifan Li, Guanwei Jiang, Xi Ou

**Affiliations:** ^1^ Peking University Shenzhen Hospital, Clinical College of Anhui Medical University, Shenzhen, China; ^2^ Department of Hepatopancreatobiliary Surgery, Peking University Shenzhen Hospital, Shenzhen, China

**Keywords:** hepatocellular carcinoma, CIBERSORT, weighted gene co-expression network analysis, bioinformatics, the cancer genome atlas

## Abstract

Hepatocellular carcinoma (HCC) is a common cancer with poor prognosis. Due to the lack of effective biomarkers and its complex immune microenvironment, the effects of current HCC therapies are not ideal. In this study, we used the GSE57957 microarray data from Gene Expression Omnibus database to construct a co-expression network. The weighted gene co-expression network analysis and CIBERSORT algorithm, which quantifies cellular composition of immune cells, were used to identify modules related to immune cells. Four hub genes (EFTUD2, GAPDH, NOP56, PA2G4) were identified by co-expression network and protein-protein interactions network analysis. We examined these genes in TCGA database, and found that the four hub genes were highly expressed in tumor tissues in multiple HCC groups, and the expression levels were significantly correlated with patient survival time, pathological stage and tumor progression. On the other hand, methylation analysis showed that the up-regulation of EFTUD2, GAPDH, NOP56 might be due to the hypomethylation status of their promoters. Next, we investigated the correlations between the expression levels of four hub genes and tumor immune infiltration using Tumor Immune Estimation Resource (TIMER). Gene set variation analysis suggested that the four hub genes were associated with numerous pathways that affect tumor progression or immune microenvironment. Overall, our results showed that the four hub genes were closely related to tumor prognosis, and may serve as targets for treatment and diagnosis of HCC. In addition, the associations between these genes and immune infiltration enhanced our understanding of tumor immune environment and provided new directions for the development of drugs and the monitoring of tumor immune status.

## Introduction

Liver cancer is one of the most common cancers in the world. The recent statistics show that the incidence of liver cancer has been increasing more rapidly than any other cancers [1]. Hepatocellular carcinoma accounts for approximately 80% of liver cancers, and cholangiocarcinoma (CCA) accounts for approximately 15%. 75% of liver cancer cases occur in Asia, and China accounts for more than half of them. Aflatoxin and chronic hepatitis B virus (HBV) infection are the two main risk factors for liver cancer in the high-incidence countries in Asia and Africa [2]. The current tests for liver cancer are AFP and PVIK II; but their diagnostic efficacies are not satisfactory, and more effective diagnostic markers are needed. Moreover, many patients already have advanced liver cancer at the time of diagnosis, and they cannot benefit from radical resection, targeted drugs or chemotherapy. This is an important factor causing poor prognosis of liver cancer [3]. Recent reports have shown that immunotherapy is a promising treatment for many advanced cancers, especially for patients with liver cancer caused by hepatitis viruses [4]. nivolumab and pembrolizumab are FDA approved drugs for treating liver cancer. These drugs open up a new direction of immunotherapy for treating advanced liver cancer; however, the objective efficiency of this treatment is only 16–20% [5], which is mainly due to the immunosuppressive property of liver tumor.

Recent studies have found that the immune checkpoint inhibitors can activate the autoimmune response to tumors by blocking immune checkpoint pathway in T cells. These immune checkpoint inhibitors showed good effects in the treatment of melanoma [6]. However, due to the immunosuppressive microenvironment of liver cancer, conventional immune checkpoint inhibitors, such as programmed death 1 (PD-1) and cytotoxic T lymphocyte-associated antigen 4 (CTLA-4), have limited therapeutic effects for liver cancer [7]. Thus, the identification of reliable biomarkers can effectively predict the therapeutic response of checkpoint inhibitors, and help monitoring the response of immunotherapy and understanding the mechanism of immune infiltration.

With the establishment of public bioinformatics databases and the advancement of bioinformatics analysis techniques, many models have been developed for identifying biomarkers. This method has been widely used to find biomarkers at the transcriptional level [8, 9]. In this study, by exploring the data from GEO public database, we identified the co-expressed genes using weighted gene co-expression network analysis (WGCNA), and examined the relationships between gene networks, phenotypes, and the expression of core genes. Moreover, we used the CIBERSORT algorithm to analyze the RNA-seq data from liver cancer patients, and identified the genes related to immune infiltration. Gene ontology (GO) and Kyoto Gene and Genome Encyclopedia (KEGG) analysis were performed to further evaluate the potential functions of the genes in key modules. Next, we analyzed the relationships between the expression of hub genes and the tumor stage, pathological classification, and patient overall survival (OS) using the TCGA database. Meanwhile, the tumor immune assessment resources (TIMER) and gene set variation analysis (GSVA) were applied to study the potential biological functions of the hub genes.

## Materials and Methods

### WGCNA Analysis

The matrix files of GSE57957 dataset were downloaded from NCBI GEO public database to extract the transcriptome data of 39 samples from liver cancer patients [10]. The weighted gene co-expression network was constructed to find the co-expression gene module and explore the associations between gene network, phenotype, and the core genes in the network. The WGCNA-R packet was used to construct a co-expression network of all genes in the dataset [11]. Then, the top 5000 genes with largest variances were screened by this algorithm for further analysis, where the soft threshold was set as 5. The weighted adjacency matrix was transformed into topological overlap matrix (TOM) to estimate network connectivity, and the hierarchical clustering method was used to construct the cluster tree of TOM matrix. Different branches of the cluster tree represent different gene modules, and different colors represent different modules. Based on the weighted correlation coefficient, genes were classified according to their expression patterns. The genes with similar patterns were grouped into one module, and all the genes were divided into several modules.

#### Immune Cell Infiltration Analysis

This study used the CIBERSORT algorithm to analyze the RNA sequencing data from Liver hepatocellular carcinoma (LIHC) patients to infer the relative proportion of 22 immune infiltrating cells [12]. We input the data of immune cell content in each patient, and then found the modular genes most relevant to immune infiltration based on WGCNA network and mRNA expression data. The specific molecular mechanisms were further explored.

#### Gene Set Enrichment Analysis

In order to obtain the biological functions and signaling pathways involved in the modules of interest in WGCNA (The yellow module has the highest correlation with regulatory T cells), we used the Metascape database (www.metascape.org) for annotation and visualization [13], and performed gene ontology (GO) analysis. Then, we analyzed the genes in a specific module and performed pathway analysis in “Kyoto Encyclopedia of Genome” (KEGG).

#### Identification of Hub Genes

We identified four central genes based on the module connectivity and the clinical characteristics of each gene in the central module. To verify the hub genes, we selected all genes in the hub module and used the Search Tool for the Retrieval of Interacting Genes (STRING; https://string-db.org/) database to construct PPI network and look for central nodes [14].

#### TCGA Data Acquisition

The TCGA database (https://portal.gdc.cancer.gov/) is currently the largest genome information database for cancers [15]. The stored data included gene expression data, miRNA expression data, copy number variation, DNA methylation, SNP, etc. We downloaded the raw mRNA expression data of the processed LIHC. There were a total of 424 samples, of which 50 were normal samples and 374 were cancer samples.

#### The Relationship Between Hub Genes and Immune Cells

The TIMER database (https://cistrome.shinyapps.io/timer/) uses RNA-Seq expression spectrum to detect immune cells infiltration in tumor tissues [16]. In this study, the relationship between the hub genes and immune cell content was analyzed using TIMER database, and the correlations between copy number and immune cells infiltration level was compared.

### Validation of Prognostic Value of the Four Genes

For external verification of the prognostic value of PA2G4, EFTUD2, GAPDH and NOP56, the transcriptome expression profiles of 202 HCC patients with complete clinical data were downloaded from the ICGC (The International Cancer Genome Consortium) (https://icgc.org/) database. We assigned patients into a high-risk and a low-risk group considering the uniform cutoff (median) and plotted the survival curve by Kaplan-Meier (K-M) method.

#### cBioPortal Database Analysis

The cBio Cancer Genomics Portal (http://cbioportal.org) is an open platform based on the TCGA database for the study of multi-dimensional Cancer genome datasets. Mutations of hub genes in HCC were analyzed using the cBioPortal tool. The OncoPrint was used to display the overview of genetic alterations in EFTUD2, GAPDH, NOP56 and PA2G4 genes in each sample.

### Gene Set Variation Analysis

Gene Set variation analysis (GSVA) is a non-parametric unsupervized method to evaluate the enrichment of transcriptome gene sets [17]. GSVA converts gene level changes into pathway level changes through comprehensive scoring of interested gene sets, and then determines the biological functional changes of each sample. In this study, we downloaded the gene sets from the Molecular Signatures Database (Version V7.0) and used GSVA algorithm to score each gene set and assess the potential biological functional changes of each sample [18].

### Statistical Analysis

All statistical analyses were performed using R 3.6. All statistical tests were bilateral; *p* < 0.05 was considered statistically significant.

## Results

**FIGURE 1:**
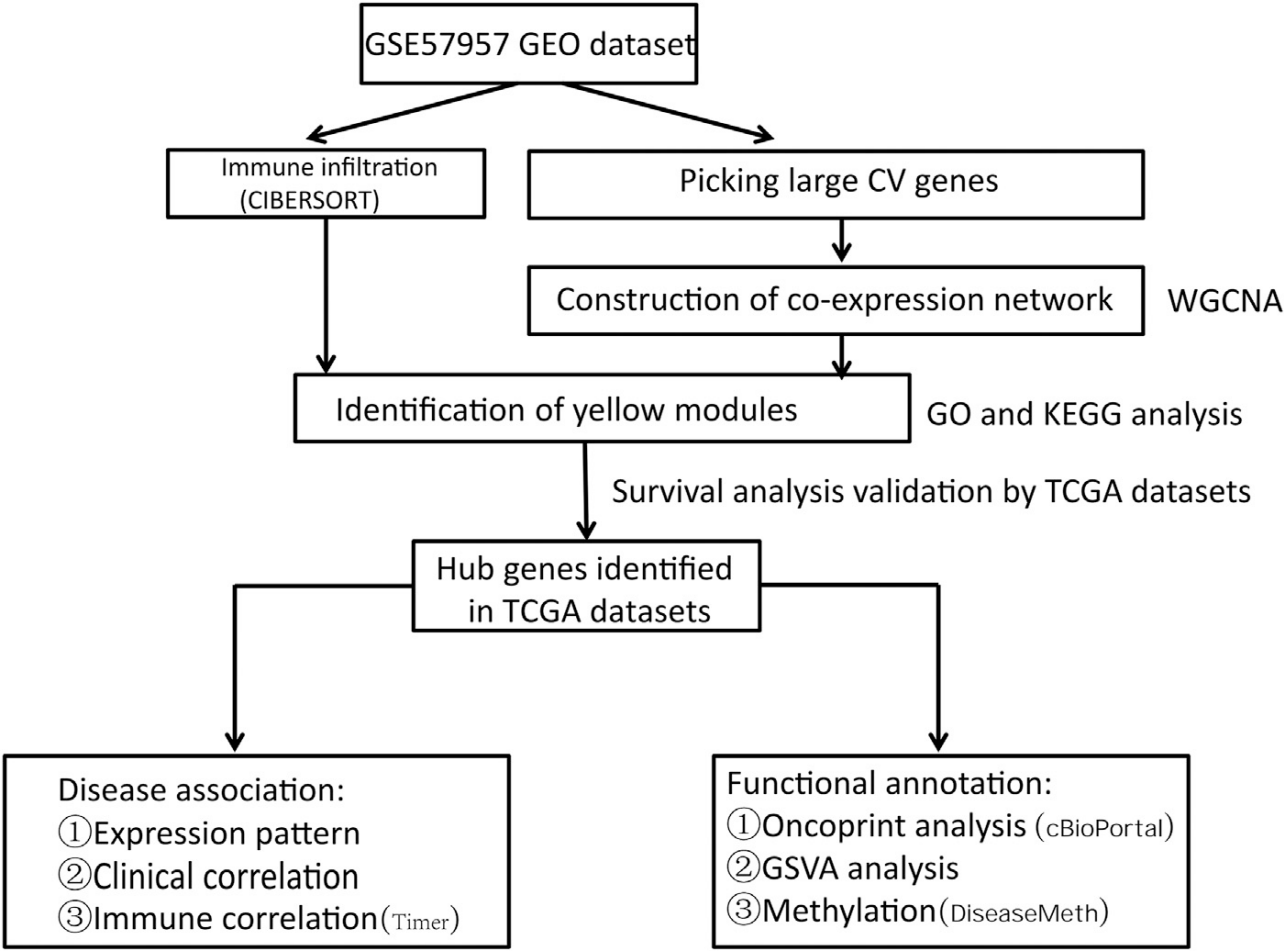
The workflow of the study. CV: Coefficient of Variation.

The research strategy is described in Figure 1.

### RNA Expression Data

We downloaded the RNA expression data of 39 HCC samples from Gene Expression Omnibus (GEO) database. The top 5000 genes with the largest variances were selected for further analysis (Supplementary Table S1).

### WGCNA and Gene Co-Expression Network for HCC

CIBERSORT is an analytical algorithm that evaluates the abundance of different cell subtypes in each sample by analyzing the RNA expression data in GEO database. We performed the R package “CIBERSORT” to obtain the relative proportions of 22 tumor-infiltrating immune cells (TIICs) in each patient sample (Supplementary Table S2), which were then used as the characteristic data for WGCNA. By performing “WGCNA,” we developed a co-expression network based on the expression levels of the 5000 genes. The average linkage and Pearson correlation values were calculated to cluster the samples in GSE57957 (Supplementary Figure S1). By setting soft-thresholding power as *β* = 5 (scale free *R* ^2^ = 0.9), we built a scale-free network (Supplementary Figures S2A,B). The dynamic hybrid cutting was used to create a hierarchical clustering tree, whose leaves represented genes. The genes with similar expression level came together and formed a branch, and 12 branches were generated to represent gene modules. Eventually, the modules with correlation coefficient greater than 0. were combined for data verification (Supplementary Figure S2C).

### Identification of Hub Modules and Enrichment Analysis

**FIGURE 2:**
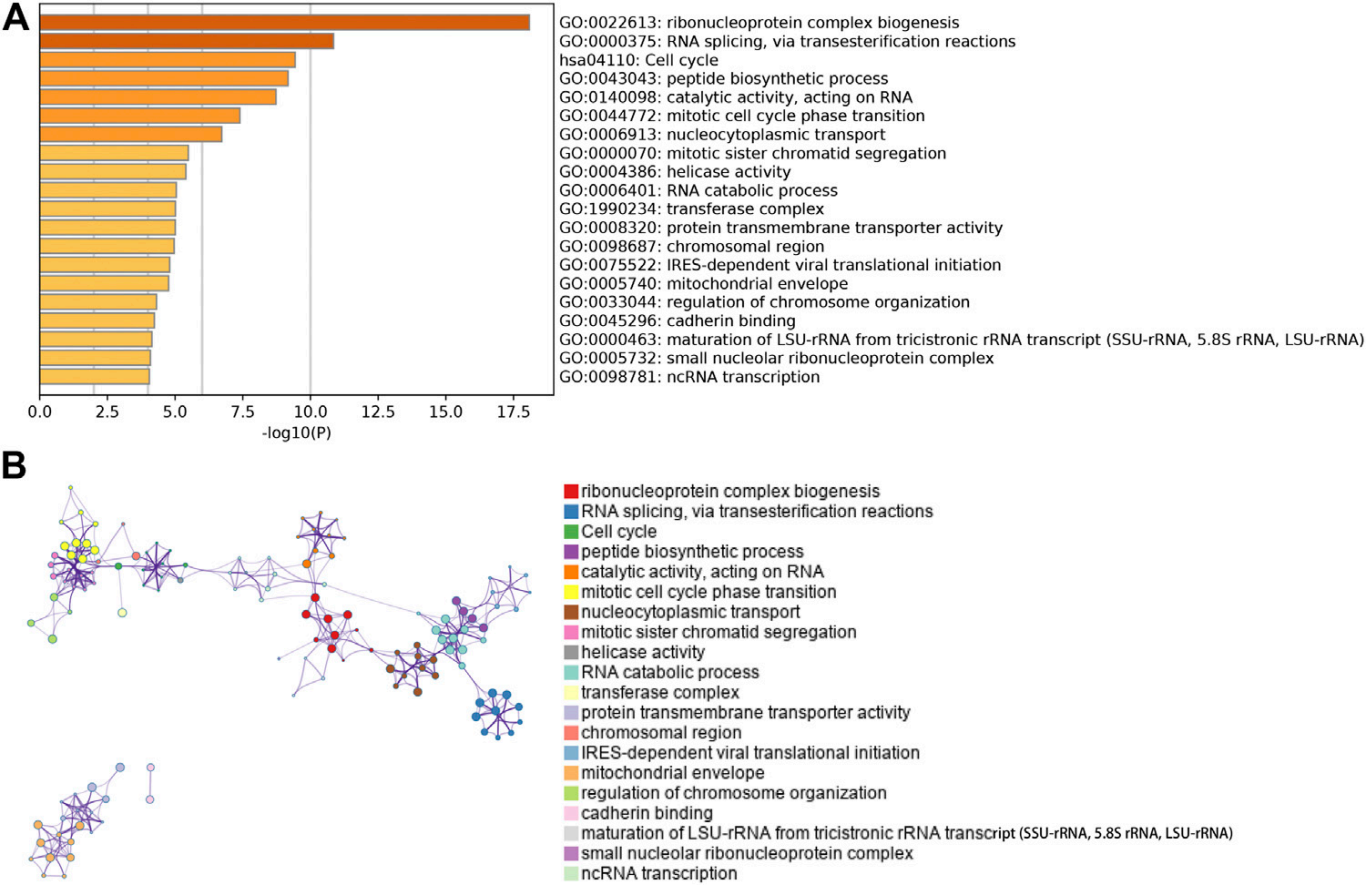
Pathway and process enrichment analysis. **(A)** Bar chart showing the first 20 enriched terms. **(B)**The network diagram of the enriched terms. Each enrichment term is a node, and the similar nodes are connected by edge. Nodes with the same cluster ID are in same color.

Among the twelve modules, the yellow module showed higher correlation than other modules for regulatory T cells (Tregs) (*R* ^2^ = 0.49, *p* = 0.002; Supplementary Figure S3A). Since the correlation between other modules and T cells was less than 0.49, we focused on the yellow module as the hub module. The genes in yellow module were analyzed using the Metascape database for pathway and process enrichment analysis. The 20 highest enrichment terms are shown in Figure 2, and the four most highly enriched terms were ribonucleoprotein complex biogenesis, RNA splicing via transesterification reactions, cell cycle, and peptide biosynthesis.

### Identification of Hub Genes

**FIGURE 3:**
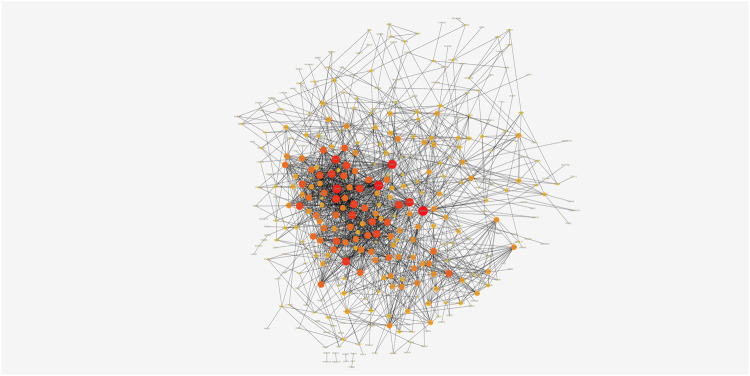
The PPI network of genes from the yellow module. The darker nodes have more edges and are more connected to other nodes.

**FIGURE 4:**
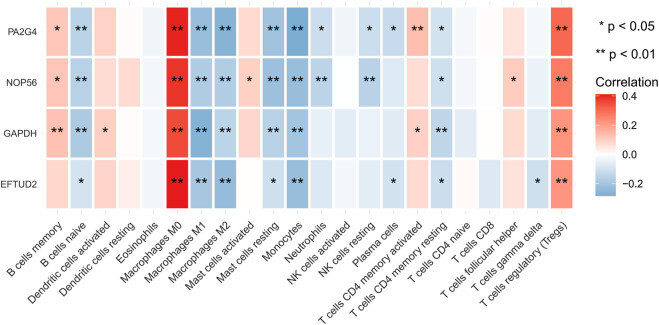
Immune correlation heatmap. The heatmap shows the correlation between the expression of four identified hub genes and the TIICs contents from TCGA database, which were quantified using the Cibersort algorithm. The red color indicates a positive correlation, and blue color indicates a negative correlation. Validation of hub genes and determination of clinical characteristics.

The highly related genes in the module were investigated as potential key factors connected to Tregs. The yellow module included a total of 440 genes (Supplementary Figure S3B). We constructed the protein-protein interaction (PPI) network for yellow module using the string database and identified five genes with the highest connectivity (Figure 3). By analyzing the relationships between the expression of these five genes and patient survival time, we selected four genes that affected the prognosis as the hub genes, which were EFTUD2, GAPDH, NOP56, and PA2G4. Next, we downloaded the correlation values between hub gene expression and the abundance of tumor-infiltrating lymphocytes from the GEO dataset, and calculated the contents of 22 immune cells using CIBERSORT algorithm. The results showed a positive correlation between the expression levels of hub genes and the abundance of tumor-infiltrating lymphocytes. As shown in Figure 4, the positive correlation values were more significant for Tregs and M0 macrophages.

### Validation of Hub Genes and Determination of Clinical Characteristics

**FIGURE 5:**
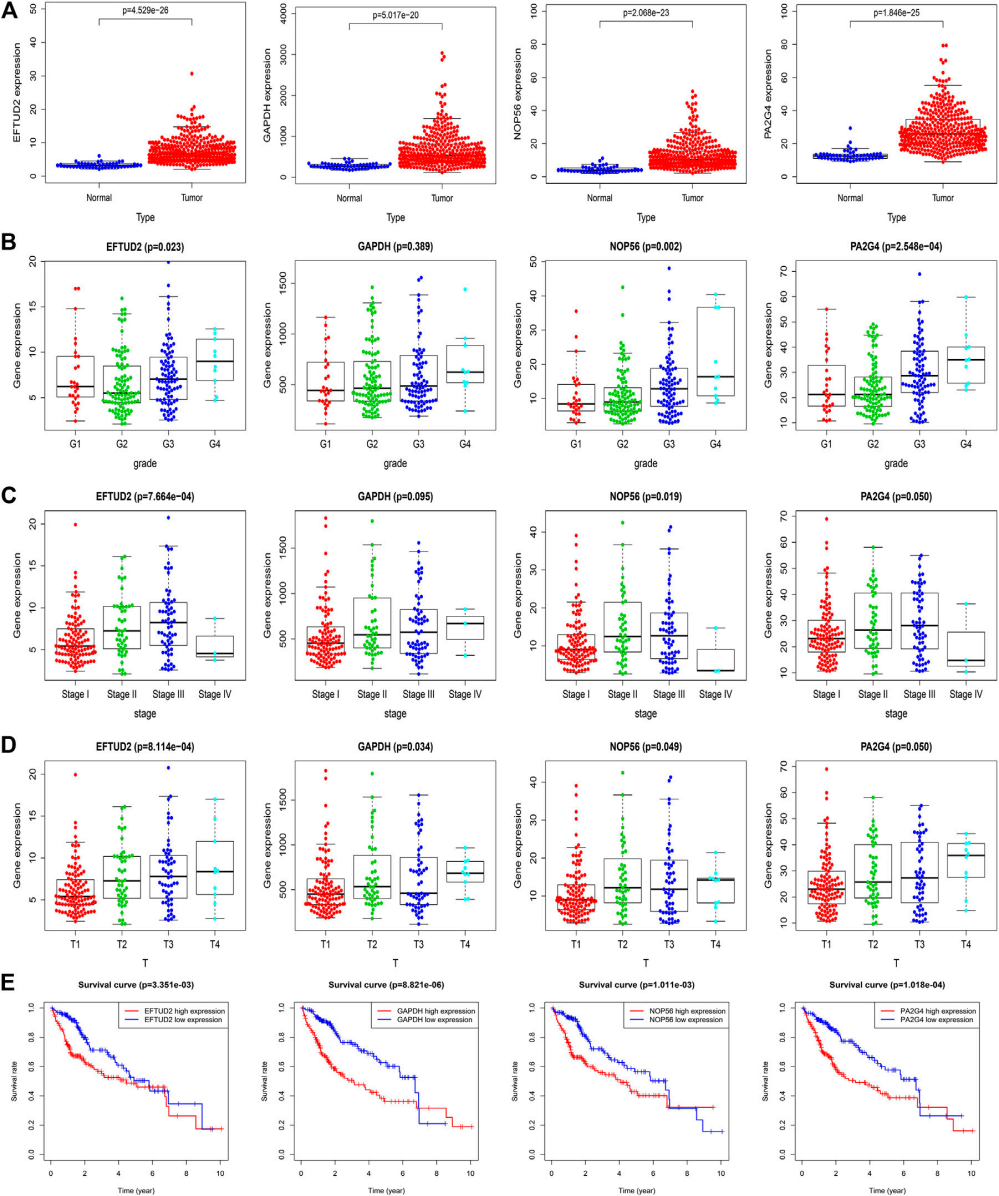
Validation of hub genes in the TCGA dataset. **(A)** The expression levels of EFTUD2, GAPDH, NOP56 and PA2G4 in tumor and adjacent normal tissues. **(B)** The expression levels of EFTUD2, GAPDH, NOP56 and PA2G4 in LIHC samples with different tumor grades. **(C)** The expression levels of EFTUD2, GAPDH, NOP56 and PA2G4 in LIHC samples with different pathological stages. **(D)** The expression levels of EFTUD2, GAPDH, NOP56 and PA2G4 in LIHC samples with different T stages. **(E)** The correlations between EFTUD2, GAPDH, NOP56 and PA2G4 expression and survival time in the TCGA dataset. The red line indicates samples with high expression level, and the blue line indicates the samples with low expression level.

We downloaded the raw RNA expression data of the four hub genes of LIHC from the TCGA database. By using Wilcoxon signed-rank test to process the data, we found that the expression levels of all hub genes were higher in tumor tissues than in normal tissues (*p* < 0.05) (Figure 5A). The correlations between hub gene expression and tumor grade, pathological stages, T stages were shown in boxplot (Figures 5B–D). The expression levels of EFTUD2, NOP56, PA2G4 were significantly positively correlated with pathological stages (*p* < 0.05). The EFTUD2 and NOP56 also showed significantly positive correlation with tumor grade and T stages. GAPDH only showed moderate correlation with pathological stages. Although no significant correlation was detected for PA2G4, its expression level showed an obvious up trend with increased pathological stages and T stages. Finally, to determine the performance of EFTUD2, GAPDH, NOP56 and PA2G4 in predicting clinical outcomes of LIHC patients, Kaplan-Meier survival curves were plotted to analyze the relationship between genes expression and patient OS (Figure 5E). The median was used as cutoff points between the high and the low-expression groups of hub genes. The results showed that the expression of EFTUD2, GAPDH, NOP56 and PA2G4 genes significantly correlated with poor overall survival.

### Validation of Prognostic Value of the Four Genes

**FIGURE 6:**
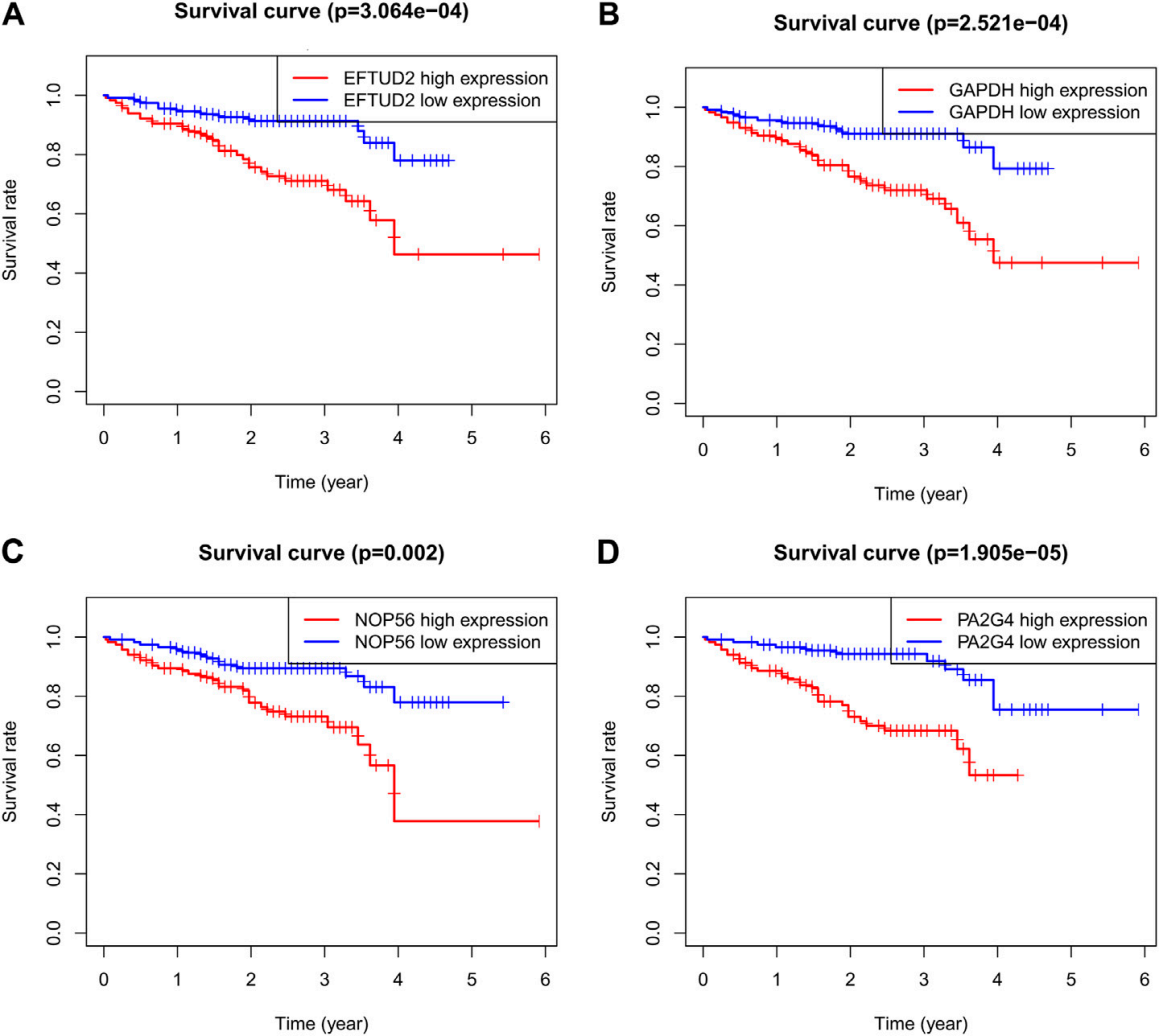
Kaplan-Meier survival curves comparing the high and low expression of **(A)** EFTUD2, **(B)** GAPDH, **(C)** NOP56, and **(D)** PA2G4 in LIHC by ICGC. Association between methylation and the expression levels of hub genes.

To validate the prognostic value of the four genes in tumors, it was downloaded and analyzed on the transcriptome expression profiles of 202 HCC patients through ICGC database to determine whether EFTUD2, GAPDH, NOP56 and PA2G4 expressions are correlated with the prognosis of LIHC. Figure 6 confirmed that high expression of these 4 hub genes affected the prognosis of LIHC.

### Association Between Methylation and the Expression Levels of Hub Genes

**FIGURE 7:**
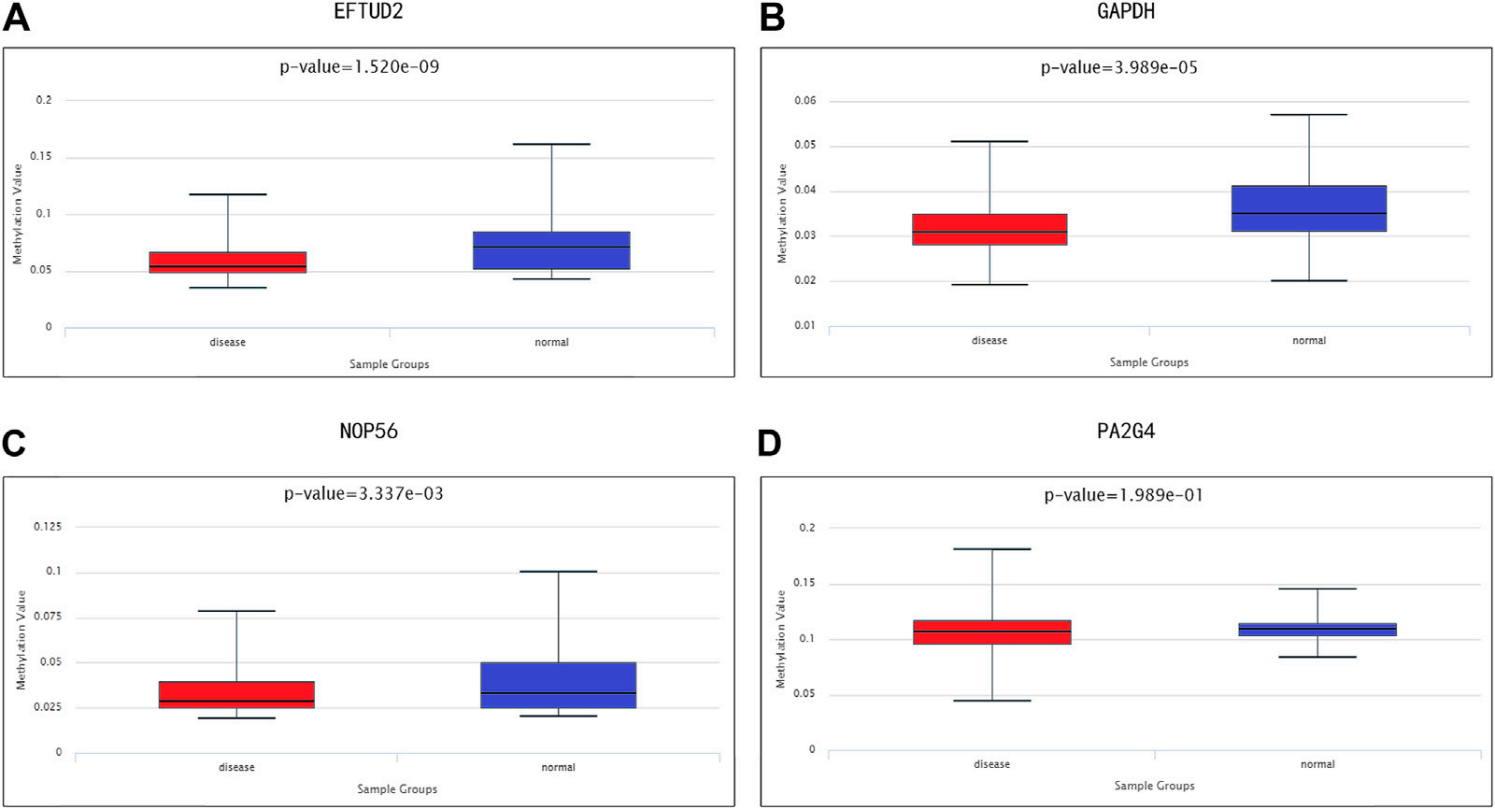
Methylation analyses of HCC hub genes. The methylation levels of **(A)** EFTUD2, **(B)** GAPDH, **(C)** NOP56, and **(D)** PA2G4 in HCC and paracancerous normal tissues were examined using DiseaseMeth 2.0. Correlation between hub gene expression and immune infiltration level in HCC.

We investigated the relationship between the expression levels of four hub genes and their methylation status to explore the underlying mechanism of the up-regulation of hub genes in HCC tissues. DiseaseMeth 2.0 analysis showed that the mean methylation levels of EFTUD2, GAPDH, NOP56 were all significantly lower in HCC compared with paracancerous normal tissues (Figures 7A–D).

### Correlation Between Hub Gene Expression and Immune Infiltration Level in HCC

**FIGURE 8:**
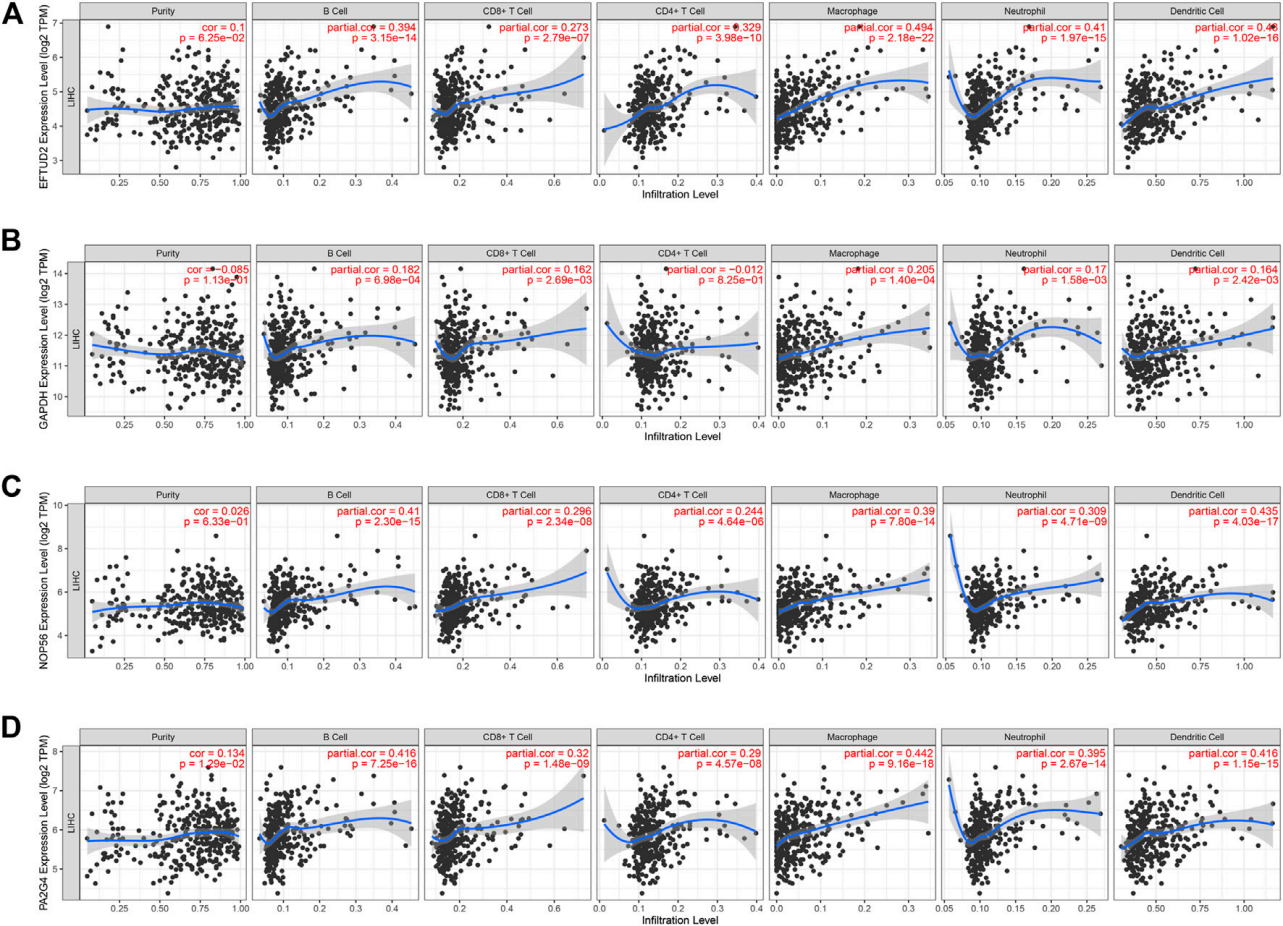
The correlation between the expression levels of hub genes and immune cell infiltration (TIMER). The correlation between the abundance of immune cell and the expression levels of **(A)** EFTUD2,**(B)** GAPDH, **(C)** NOP56, and **(D)** PA2G4. Each dot represents a sample in the TCGA dataset.

**FIGURE 9:**
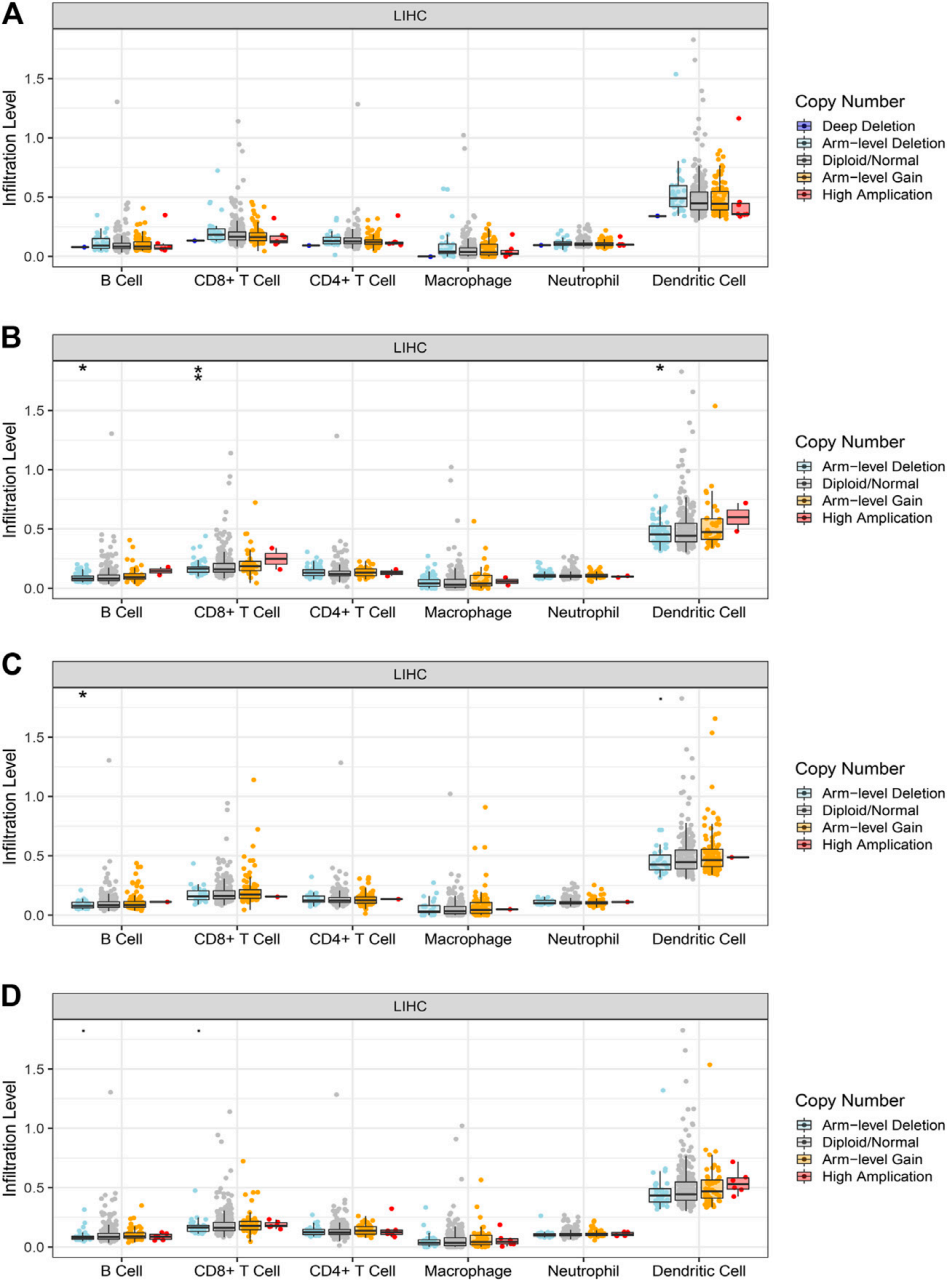
The correlations between the copy number of hub genes and immune cells infiltration level in LIHC. The correlation between the infiltration level of immune cell and copy number of **(A)** EFTUD2,**(B)** GAPDH, **(C)** NOP56, and **(D)** PA2G4. GAPDH affects the infiltrating levels of B cells, CD8^+^ T cells and dendritic cells in HCC. NOP56 affects the infiltrating levels of B cells in HCC(* <0.05, ** <0.01). Genomic alterations of hub genes in HCC.

We performed a comprehensive investigation on the correlations between the expression levels of EFTUD2, GAPDH, NOP56, PA2G4 and immune cell infiltration using the TIMER database. Interestingly, EFTUD2, GAPDH, NOP56 and PA2G4 were all positively associated with B cells, CD8^+^ T cells, neutrophils, macrophages, and dendritic cells levels in tumors. Except for NOP56, the other three genes showed positive correlation with CD4^+^ T cells. On the other hand, no or weak associations were observed between the levels of these four hub genes and tumor purity (Figures 8A–D). These results showed that the expression levels of EFTUD2, NOP56, PA2G4 were significantly correlated with the dominant immune cells infiltration levels. Finally, The correlations between copy number and infiltration levels were plotted using boxplot (Figures 9A–D). In particular, GAPDH had significant correlations with the infiltrating levels of CD8^+^ T cells, B cells, and dendritic cells.

### Genomic Alterations of Hub Genes in HCC

**FIGURE 10:**
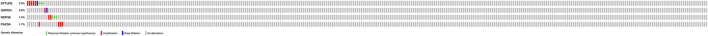
Genomic alterations of hub genes in HCC. EFTUD2 is the most frequently mutated gene. Four hub genes are altered in 19 samples (5%). GSVA Analysis for hub genes.

Based on DNA sequencing data from LIHC patients, we determined the types and frequencies of EFTUD2, GAPDH, NOP56, and PA2G4 genetic alterations in HCC using the cBioPortal online tool. Given the important clinical implications of these hub genes, we investigated the genetic alterations of EFTUD2, GAPDH, NOP56, and PA2G4 and found that amplification was the most common change for these genes (Figure 10). A total of 19 samples had mutations in these genes and EFTUD2 was the most frequently mutated gene (2.5%).

### GSVA Analysis for Hub Genes

**FIGURE 11:**
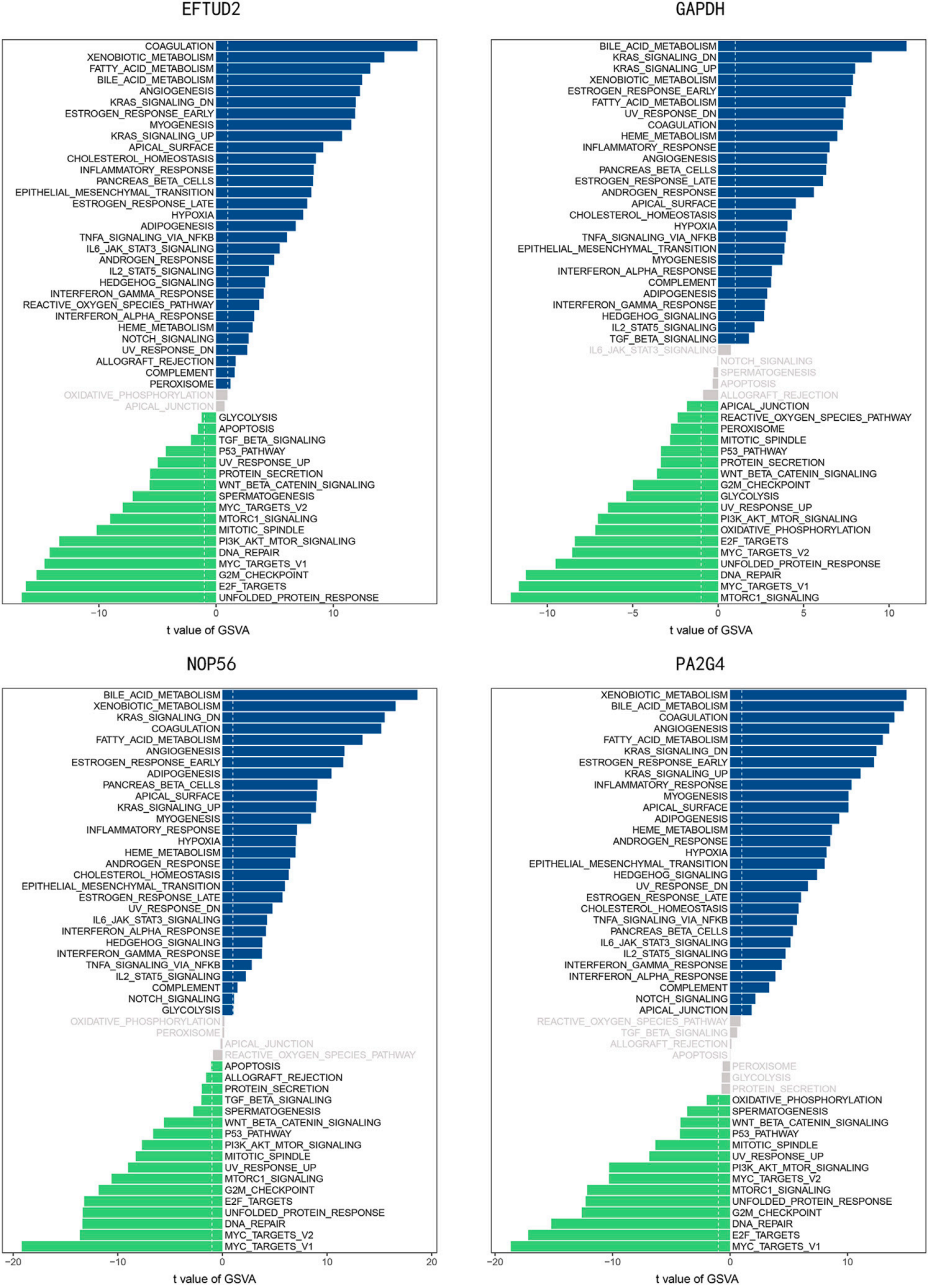
The GSVA analysis for four hub genes.

Gene set variation analysis was performed for the four hub genes. As shown in Figure 11, several hallmark gene-sets, including “ANGIOGENESIS,” “KRAS SIGNALING UP,” “P53 PATHWAY,” “PI3K AKT MTOR SIGNALING,” “EPITHELIAL_MESENCHYMAL_TRANSITION (EMT),” “MYC TARGETS V1,” were observed in all four hub genes.

## Discussion

Surgery is a common treatment method for early liver cancer, but its effect is not satisfactory. Studies have shown that most patients with HCC will eventually need palliative care [19, 20]. On the other hand, sorafenib, which is a first-line drug for HCC patients, has limited benefit for patient survival. Thus, immune checkpoint therapy, and new tumor biomarkers or therapeutic targets have become the focus of recent liver cancer research [21]. For example, Hu X used similar methods to confirm that YWHAB, PPAT, and NOL10 are novel biomarkers and to verify their diagnostic and prognosis value for HCC [22]. In this study, by using bioinformatics and comprehensive analyses, we identified hub genes that were confirmed to be prognosis factors for HCC. Furthermore, these genes were strongly correlated with immune cell infiltration in HCC.

We used the content of immune cells and mRNA expression data from HCC patients to construct the WGCNA network and identified the gene module most related to immune infiltration. Next, we identified the genes that were most closely linked in the co-expression network and the protein-protein network, including EFTUD2, GAPDH, NOP56, and PA2G4. Furthermore, we downloaded the data from TCGA to analyze the expression levels of these genes and their clinical characteristics. The results showed that these four genes were overexpressed in liver cancer tissues, and EFTUD2, GAPDH, NOP56 had high methylation levels in normal tissues, indicating that these genes could be used as biomarkers for liver cancer. We then explored the relationships between the expression levels of these genes and the tumor stage, tumor grade, and survival time, which are important properties for potential prognostic factors. By investigating TIMER/STRING database, we found positive correlations between the expression levels of four hub genes and immune cell infiltrating levels, particularly for T cells. However, the results of CIBERSORT showed that CD8^+^ T cells and neutrophils infiltrated less in each gene module, which was different from the TIMER database, resulting from the difference between the two algorithms. CIBERSORT quantified 22 kinds of immune cells, but it was different from TIMER which only quantified six kinds of immune cells. TIMER does not normalize the predicted value to 1, so the results cannot be interpreted as cell fraction or compared in different data sets. Also, many signaling pathways, such as ANGIOGENESIS, P53 PATHWAY, EPITHELIAL MESENCHYMAL TRANSITION (EMT) and MYC TARGETS V1, were enriched in the groups with upregulated expression level of these hub genes, suggesting their contribution to the occurrence, progression and tumor microenvironment of HCC. These analyses suggest that EFTUD2, GAPDH, NOP56, and PA2G4 were potential biomarkers for the diagnosis and prognosis of hepatocellular carcinoma.

There have been a lot of studies showing that these four hub genes are involved in tumor development, and recent studies have shown that several of them are involved in liver cancer. EFTUD2 is a mRNA splicing regulator, and its mutation causes a multiple malformation syndrome termed as mandibulofacial dysostosis with microcephaly. EFTUD 2 mainly restricts HCV infection through a melanoma differentiation-associated protein 5 (MDA5)-mediated JAK-independent pathway. Study has shown that EFTUD2 plays an important role in innate immune response to virus infection [23]. Zhonglin Lv et al. found that myeloid loss of EFTUD2 led to impaired activation of macrophage NF-κB and ERK, which reduced the release of pro-inflammatory cytokines and growth factors, and ultimately inhibited tumor development and progression [24]. A recent study showed that the expressions of COPZ1 and EFTUD2 were significantly higher in tumor tissues compared with normal tissues and both two genes may correlated with poor prognosis of liver cancer based on the TCGA database [25]. GAPDH is regulated by several cancer-related factors, and its overexpression is an event downstream of p53. GAPDH overexpression is involved in the processes where cancer cells hijack normal pathways [26, 27]. Recent evidence suggests that GAPDH plays an important role in tumor cell survival, tumor angiogenesis, gene expression control in tumor cells, and post-transcriptional regulation of mRNA in tumor cells [28]. Yihang Gong et al. showed that intranuclear GAPDH was involved in hypoxia-induced HSCs apoptosis, which could inhibit tumor growth and repress HCC progression [29]. Feiwen Deng proposed a scoring system based on SLC2A1, ENO1, LDHA and GAPD with sustained predictive ability for overall survival in HCC patients [30]. NOP56 is part of the box C/D small nucleolar RNAs (snoRNAs). It can regulate the posttranscriptional modification of ribosomal RNAs and can act as either a tumor suppressor or an oncogene [31]. Recent study shows that NOP56 overexpression is an antecedent event in the relapse of B-cell precursor acute lymphoblastic leukemia [32], and it has been shown to be a potential prognostic marker for metastatic renal cell carcinoma [33]. PA2G4 belongs to the DNA/RNA binding protein family and has been shown to play a role in cell growth, apoptosis and differentiation [34]. Jessica Koach et al. found that PA2G4 was an oncogene in human, mice, and zebrafish, and might be a new target for designing MYCN inhibitors [35].

In conclusion, this study identified four hub genes, EFTUD2, GAPDH, NOP56, and PA2G4, which were overexpressed in tumors and had good prognostic efficacy for HCC. All these four genes were correlated with the infiltration of immune cells in HCC patients, and the biological pathways of these genes are involved in HCC development. These findings may provide a new perspective for further understanding of the development of HCC and new directions for the treatment of HCC. However, this study has certain limitations. Additional samples are required to confirm these results, and the regulatory mechanism of these genes in HCC requires further investigation.

## Data Availability

The datasets presented in this study can be found in online repositories. The names of the repository/repositories and accession number(s) can be found in the article/Supplementary Material.

## Abbreviations

Bioinformatics analysis; CIBERSORT, Cell-type identification by estimating relative subsets of RNA transcripts; GEO, Gene Expression Omnibus; GSVA, Gene Set variation analysis; HCC: Hepatocellular carcinoma; PPI: Protein-protein interactions; TCGA, The Cancer Genome Atlas; TIICs: Tumor-infiltrating immune cells; WGCNA: Weighted gene co-expression network analysis
