# Supplementary figures and images for: Identification of Biomarkers Related to Immune Cell Infiltration in Hepatocellular Carcinoma Using Gene Co-Expression Network
Source: Pathol Oncol Res. 2021 Apr 2;27:601693. doi: 10.3389/pore.2021.601693 (PMC8262220; doi:10.3389/pore.2021.601693)

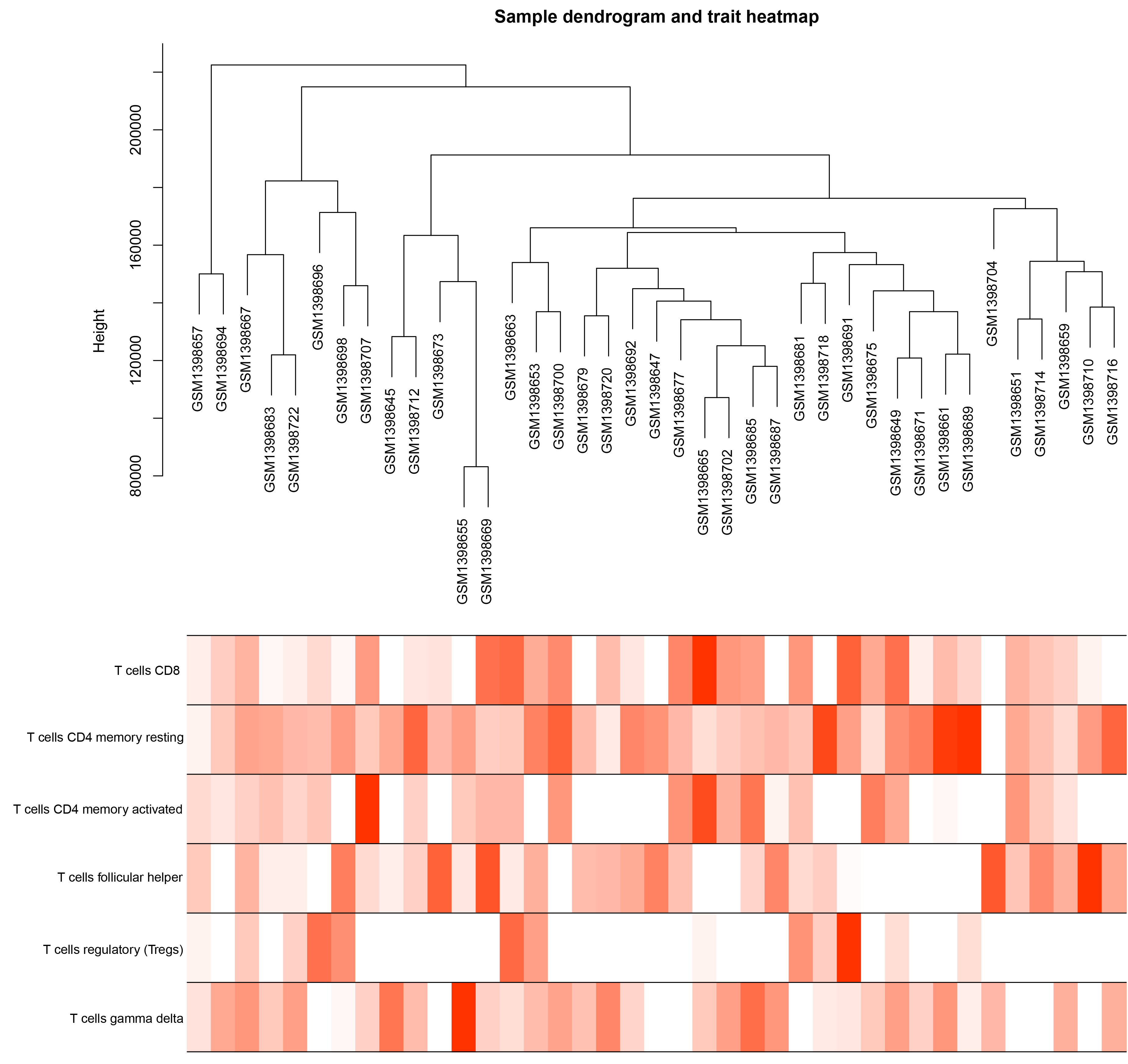

Supplement: Supplementary file 1 [file Image1.TIFF]

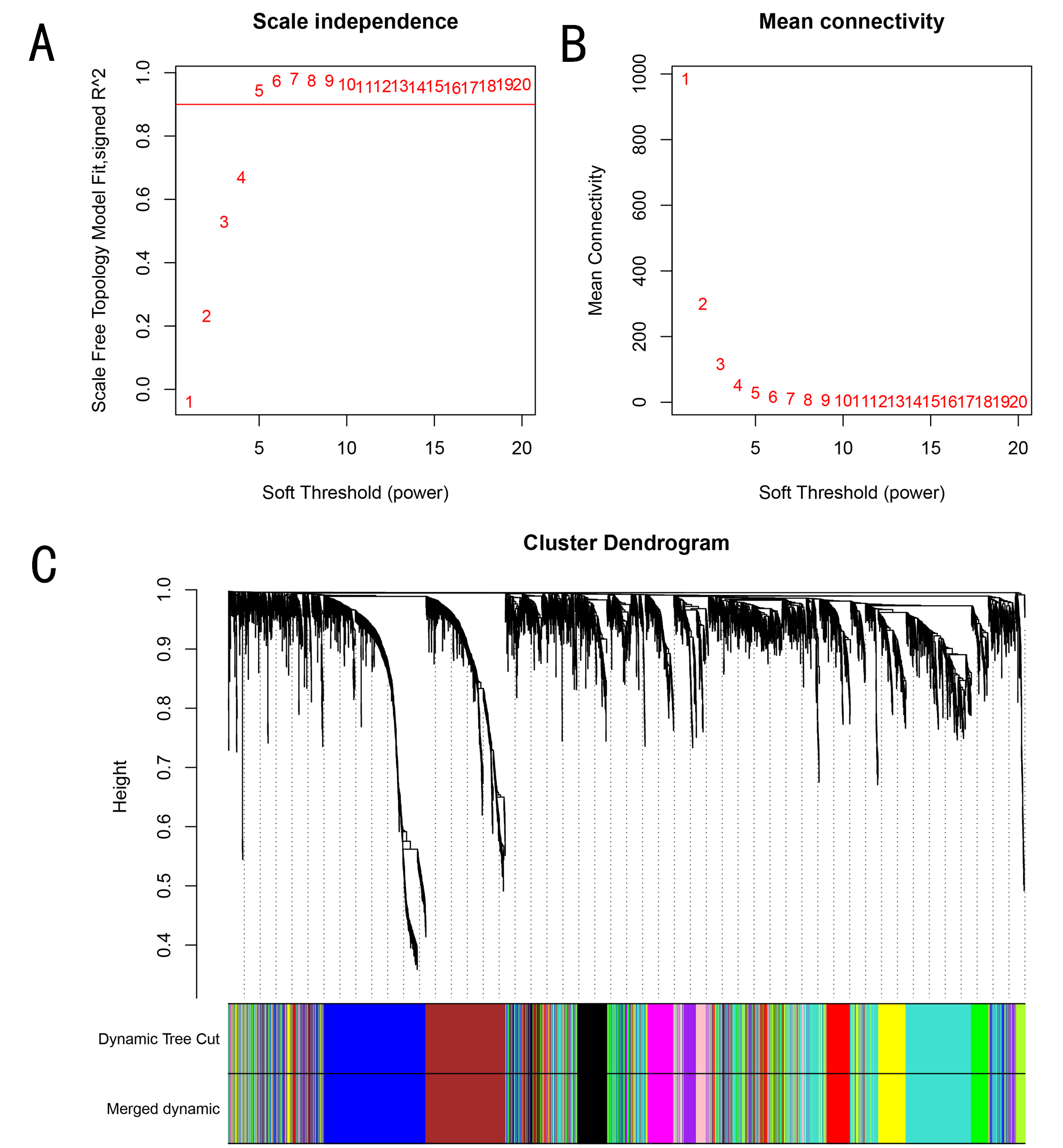

Supplement: Supplementary file 2 [file Image2.TIF]

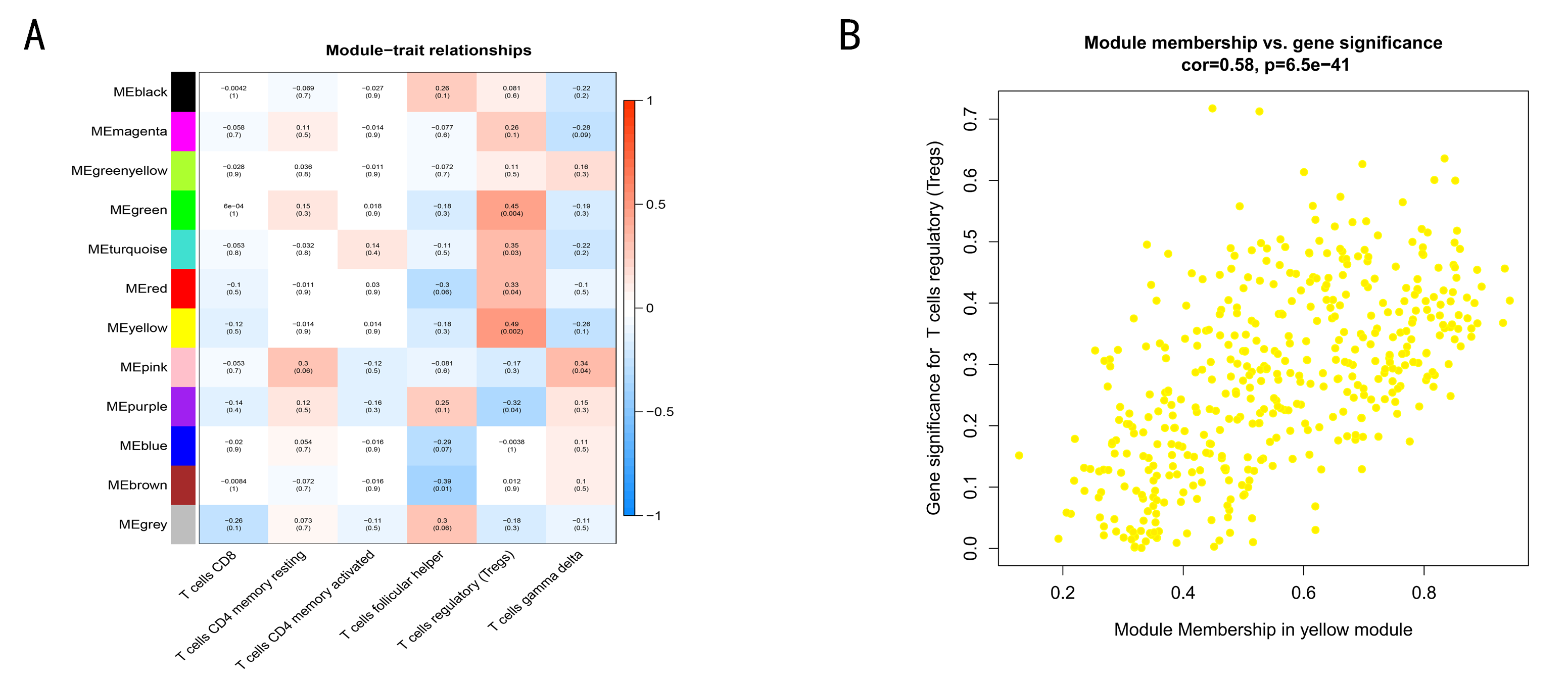

Supplement: Supplementary file 3 [file Image3.TIF]
